# Approach to amoebic colitis: Epidemiological, clinical and diagnostic considerations in a non-endemic context (Barcelona, 2007-2017)

**DOI:** 10.1371/journal.pone.0212791

**Published:** 2019-02-21

**Authors:** Sílvia Roure, Lluís Valerio, Laura Soldevila, Fernando Salvador, Gema Fernández-Rivas, Elena Sulleiro, Míriam Mañosa, Nieves Sopena, José Luis Mate, Bonaventura Clotet

**Affiliations:** 1 Infectious Disease Department, North Metropolitan International Health Unit PROSICS, Germans Trias i Pujol University Hospital, Universitat Autònoma de Barcelona, Badalona, Spain; 2 North Metropolitan International Health Unit PROSICS, Institut Català de la Salut, Santa Coloma de Gramenet, Barcelona, Spain; 3 Infectious Disease Department, Vall d’Hebron University Hospital, PROSICS Barcelona, Universitat Autònoma de Barcelona, Barcelona, Spain; 4 Microbiology Depatment, Clinical Laboratory North Metropolitan Area, Germans Trias i Pujol University Hospital, Departament of Genetics and Microbiology, Autonomous University of Barcelona, Badalona, Spain; 5 Microbiology Department, Vall d’Hebron University Hospital. PROSICS Barcelona, Universitat Autònoma de Barcelona, Barcelona, Spain; 6 Inflammatory Bowel Disease Unit, Gastroenterology Department,Germans Trias i Pujol University Hospital and Centro de Investigaciones Biomédicas en Red de Enfermedades Hepáticas y Digestivas, Badalona, Spain; 7 Infectious Diseases Department, Germans Trias i Pujol University Hospital, Universitat Autònoma de Barcelona, Badalona, Spain; 8 Department of Pathological Anatomy, Germans Trias i Pujol University Hospital, Badalona, Spain; 9 Infectious Disease Department, AIDS Research Institute-IrsiCaixa, Germans Trias i Pujol University Hospital, Universitat Autònoma de Barcelona, Badalona, Spain; University of Witwatersrand/NHLS, SOUTH AFRICA

## Abstract

**Background:**

Amoebic colitis is the most frequent clinical manifestation of invasive intestinal infection due to *Entamoeba histolytica* and a common cause of diarrhoea worldwide. Since higher transmission rates are usually related to poor health and exposure to unhygienic conditions, cases reported in Europe usually involve immigrants and international travellers. The goal of this study was to characterise both the clinical and the epidemiological features of a European population diagnosed with amoebic colitis and then to evaluate the diagnostic tools and therapeutic options applied.

**Methods and results:**

This was a retrospective observational study in which data from all patients diagnosed with amoebic colitis attending at the International Health Units of two tertiary referral hospitals, Germans Trias i Pujol University Hospital (Badalona, North Barcelona Metropolitan Area) and Vall d’Hebron University Hospital (Barcelona city) between 2007 and 2017 were analysed. During the study period 50 patients were diagnosed with amoebic colitis. Thirty-six (72%) were men, and immigrants accounted for 46% of all cases. Antecedents of any international travel were reported for 28 (56%), the most frequent destinations having been the Indian subcontinent, South and Central America and sub-Saharan Africa. Preexisting pathological conditions or any kind of immunosuppression were identified in 29 (58%) patients; of these, 13 (26%) had HIV infection—all of them men who have sex with men—and 5 (10%) had inflammatory bowel disease. Diarrhoea, abdominal pain and dysentery were the most frequently recorded symptoms of invasive amoebae. Diagnosis was made through microbiological study in 45 (90%) and/or histological identification of amoebae in colon biopsies in 10 (20%). After treatment with metronidazole (82%) or tinidazole (8%), all patients had good outcomes. Post-acute intraluminal treatment was indicated in 28 (56%).

**Conclusions:**

Amoebic colitis should be suspected in patients with diarrhoea and compatible epidemiological risk factors (immigration, travelling abroad or men who have sex with men), especially if some degree of immunosuppression concurs. These risk factors must be taken into account in any diagnostic approach to inflammatory bowel disease (IBD), and active searches for stool parasites should be performed in such cases to rule out misdiagnosis or simultaneous amoebic infection. Treatment should include intraluminal anti-amoebic treatment in order to avoid relapse and prevent further spread of the disease.

## Introduction

Intestinal amoebiasis is caused by the protozoon *E*. *histolytica*. Approximately 50 million people develop colitis or extraintestinal disease worldwide as a result of *E*.*histolytica* infection, with over 100,000 deaths reported annually [1]. Prevalence is disproportionately higher in developing countries because of poor socioeconomic and sanitation conditions. Areas with the highest rates of amoebic infection include India, Africa, Mexico, and some parts of Central and South America. In developed countries, therefore, amoebiasis is generally seen in migrants and travellers coming from endemic areas [2].

Infection by *E*. *histolytica* starts with the ingestion of mature cysts from fecally-contaminated food or water. Once in the intestinal lumen, excystation takes place and trophozoites develop. These use a galactose- and N-acetyl-D-galactosamine (Gal/GalNAc)-specific lectin to adhere to colonic mucins and thereby penetrate the mucous layer of the large intestine [3].

Interaction of the parasite with the intestinal epithelium causes an inflammatory response. In some patients extraintestinal dissemination may occur and trophozoites reach the peritoneum, liver and other areas. Factors controlling invasion probably include whether the parasite senses a “quorum” as signalled by the Gal/GalNAc-specific lectin, its interactions with the intestinal bacterial microbiota, and the innate or acquired immune responses of the host.

Though most infections are asymptomatic (90% of humans harbouring the parasite are asymptomatic carriers), the most common clinical manifestations include dysentery and extraintestinal disease [2]. Patients with amoebic colitis typically present with a several-week history of cramping abdominal pain, weight loss and watery or bloody diarrhoea. Differential diagnosis should include other infectious and noninfectious diseases such as IBD. Most patients with symptoms have a clinical course similar to chronic colitis, but some present symptoms of acute colitis even months to years after exposure [4]. The most pathological host response to amoebic infection is fulminant necrotising colitis and perforation, a complication observed in approximately 0.5% of cases. Associated mortality rates can reach around 40% [5]. High-risk populations for developing invasive amoebiasis include infants, pregnant women, and patients who are taking immunosuppressive drugs, especially patients receiving corticosteroids [6].

The diagnosis of *E*. *histolytica* infection is challenging, and current methods lack sensitivity. In developing countries, intestinal amoebiasis is commonly diagnosed by identifying cysts or motile trophozoites by wet mount examination of stool samples. The drawbacks of this method include its low sensitivity and specificity, with false positive results common owing to the presence of *E*. *dispar* or *E*. *moshkovskii*. Ideally, diagnosis should be based either on the detection in stool specimens of *E*. *histolytica*-specific antigen/DNA or the presence of anti-amoebic antibodies in serum [2,7]. Serology is most useful in patients with extraintestinal disease when organisms are not found in stool samples. Antigen detection may be useful as an adjunct to microscopic diagnosis and can distinguish between pathogenic and nonpathogenic amoebas. Examination of colonic mucosal biopsy specimens and exudates can reveal a wide variety of histopathological findings associated with amoebic colitis. These include diffuse, nonspecific, mucosal thickening with or without ulceration and, in rare cases, the presence of amoebas in the mucinous exudate; focal ulcerations with or without amoebas in a diffusely inflamed mucosal layer; classic flask-shaped lesions with ulceration extending through the mucosa and muscularis mucosa into the submucosa; and necrosis and perforation of the intestinal wall [8].

The increasing popularity of international travel as well as the presence of a growing immigrant population in the greater Barcelona area has led to an increase in amoebic colitis cases and a subsequent rise in the diagnostic challenges facing the local health services.

The objective of the present study was therefore to characterise both clinical and epidemiological aspects of a population diagnosed with the disease in the Barcelona area as well as to evaluate the diagnostic tools and therapeutic approaches applied to them.

## Materials and methods

### Study population

From January 2007 to December 2017, sentinel clinicians from the North Metropolitan Imported Diseases Working Group consecutively recorded all cases of individuals diagnosed with amoebic colitis at two tertiary referral hospitals with advanced diagnostic capability in the greater Barcelona area (Germans Trias i Pujol Hospital and Vall d’Hebron Hospital). These institutions belong to the Catalan public health service and the medical services they provide are therefore easily accessible and free of charge.

Individuals were considered confirmed cases of amoebic colitis when they met one of the following criteria: a) presence of colitis and evidence of cysts or trophozoites of *E*. *histolytica/E*. *dispar* in stool samples; b) presence of colitis and *E*. *histolytica* antigen detected in stool samples; or c) presence of cysts or trophozoites in microscopy scrapings or colon biopsy. A serological test was performed to study extraintestinal amoebic disease.

The following variables were assessed: age, sex, immigrant (yes/no), length of residence in the European Union (years), presence or absence of recent international travel, length of trip (days), pre-travel health advice (yes/no), immunodeficiency (HIV+, corticosteroids and/or immunosuppressive treatment), presence of IBD (Crohn’s disease/ulcerative colitis; yes/no), clinical presentation (fever, diarrhoea, dysentery, abdominal pain, weight loss, abscess), diagnostic approach (stool microscopy, antigen testing, serology, suggestive colonoscopy findings, biopsy), treatment (metronidazole, tinidazole, intraluminal treatment), new IBD diagnosis (yes/no), and wrong IBD diagnosis (yes/no).

Data was collected and analysed anonymously in compliance with the usual confidentiality requirements. Written informed consent had been obtained from all patients or their guardians at the time they were assessed. This study was approved by the clinical research ethics committee of the Germans Trias Hospital (authorization no. PI-17-259).

### Diagnostic tests and treatment

Diagnostic testing of patients with suspected colitis depended on the sample obtained. In the case of stool samples, feces were fixed with formalin. The stool samples were immediately fixed after their emission. This fact means that any type of parasitic structure, both cyst and trophozoite, is adequately preserved for subsequent microscopic examination. Samples were sent to the respective microbiology departments, where wet mount examination was used to identify possible cysts and/or trophozoites after ethyl-acetate concentration. In some cases, to confirm the results, fresh stool samples were tested for anti-amoebic antigens using the Entamoeba CELISA Path® test (Cellabs, Brookvale, New South Wales, Australia). In a few cases, diagnosis was based on biopsies from endoscopy analysed at the respective Pathology Departments. A serological test was performed to study extraintestinal disease by using the NovaLisa Entamoeba histolytica IgG (NovaTec Immunodiagnostica GmbH. Dietzenbach, Germany).

Invasive colitis was treated with metronidazole 750 mg PO bid × 7 to 10 days or tinidazole 2 g PO once × 3 days, followed by a luminal agent to eliminate intraluminal cysts, generally paromomycin 25 to 30 mg/kg/d PO in 3 doses × 7 days.

### Statistical analysis

Categorical variables and continuous data were reported as percentages and mean ± standard deviation (SD), respectively. To detect significant differences between categorical variables, the Chi-squared test was performed with Fisher’s correction if needed. For continuous variables the Student-t test or the Mann–Whitney U test, when appropriate, was used instead. Statistical significance was established at α = 0.05. All reported p values are two-tailed. Statistical Package for the Social Science for Windows (SPSS, version 20; Chicago, Illinois, USA) was used for the statistical analysis.

## Results

During the ten-year study period, 50 individuals were diagnosed with amoebic colitis. Their socio-demographic and clinical characteristics are displayed in Table 1. Twenty-eight (56%) had a history of international travel, the Indian subcontinent, South and Central America and sub-Saharan Africa being the most frequent destinations. Thirteen (26%) of the patients had HIV infection, all of them were men who have sex with men (MSM), of whom four (8%) lacked antecedents of travel abroad. Ten (20%) individuals had been initially diagnosed as having IBD but this diagnosis was found to be wrong for five of these (10%) after reassessment confirmed amoebic colitis. Concurrent IBD and amoebic colitis was confirmed in five cases (10%).

**Table 1:** Socio-demographic and clinical characteristics of amoebic colitis cases (n = 50).

VARIABLE		N	%
Sex			
	Male	36	72
**Mean age in years (range)**		**40 (22–74)**	
**Origin**			
	Local	27	54
	Immigrant	23	46
**Prior international travel**			
	Yes	28	56
	No	22	44
**Destination of prior travel**			
	Indian subcontinent	9	18
	South America	5	10
	Sub-Saharan Africa	5	10
	Southeast Asia	3	6
	Central America	5	10
	North Africa	1	3
**Duration of prior travel**			
	< 15 days	1	2
	15–30 days	9	18
	30–90 days	8	16
	> 90 days	7	14
**Had received pre-travel health advice**			
	No	35	70
**Pathological conditions**			
	HIV	13	26
	Neoplasia	2	4
	Renal insufficiency	2	4
	Inflammatory bowel disease	5	10
	Diabetes mellitus	1	2
	Corticosteroids treatment	6	12
**When symptoms manifested (data for only 25 subjects)**			
	During trip	9	36
	After trip	16	64
**Symptoms**			
	Fever	12	24
	Diarrhoea	45	90
	Dysentery	19	38
	Abdominal pain	31	62
	Weight loss	11	22
**Extraintestinal amoebiasis**			
	Abscess	8	16

Diarrhoea, abdominal pain and dysentery were the most frequent symptoms. Bivariate analysis showed that individuals with travel antecedents tended to report higher pain scores (p = 0.033) and were more likely to have dysentery (p = 0.09).

**Fig 1:**
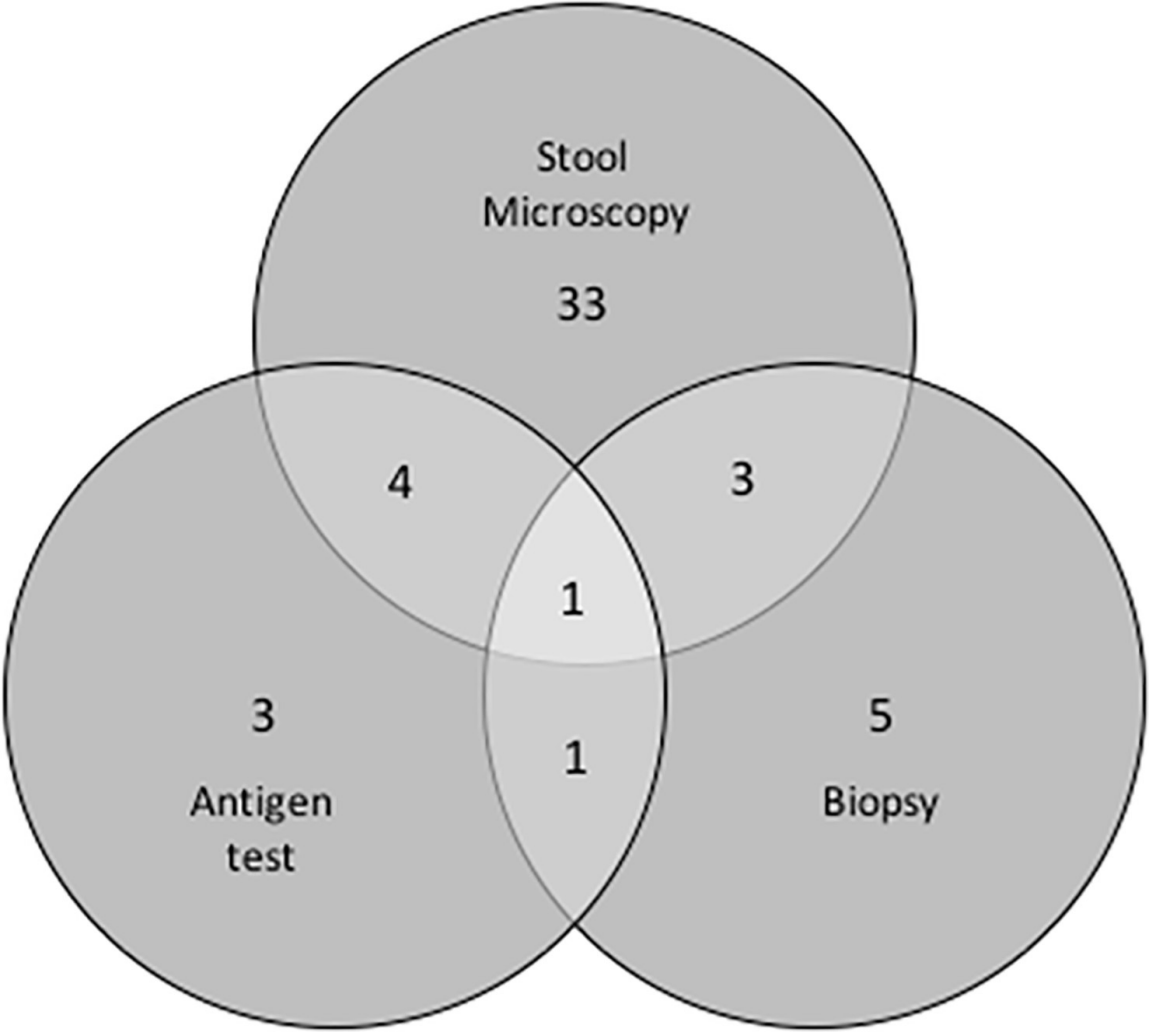
Venn diagram showing diagnostic tests and the number of subjects on whom each of these tests was performed (total number = 50). Positive stool microscopy 41/45 (91%), positive antigen test 9/12 (75%), positive biopsy 10/18 (55. 5%).

As detailed in Fig 1, diagnosis was based on microbiological examination for 45 (90%) and/or histological visualization of trophozoites after colon biopsy in 10 (20%). All evolved favourably after starting treatment with metronidazole (70%) or tinidazole (10%). Luminal treatment with paromomycin, diiodohydroxyquin or diloxanide furoate was indicated in 16 instances (53.3%).

Of patients who had inflammatory bowel disease, 4 had ulcerative colitis and 1 Crohn's disease. Two of them were diagnosed with inflammatory bowel disease and amoebic colitis at the same time and three had a previous diagnosis of inflammatory bowel disease. They were more likely to be female (p = 0.018), have dysentery symptoms (p = 0.007) and yield negative stool examination results (p = 0.048).

Five individuals (10%) were initially misdiagnosed as having IBD (see Table 2). In three cases, the final diagnosis of amoebic colitis did not occur until more than two years had elapsed and, in the meantime, these three patients received corticosteroids and other immunosuppressive treatments. One of them developed an amoebic liver cyst three years after the onset of colitis, while receiving the immunosuppressive treatment.

**Table 2:** Cases of wrong IBD diagnosis.

CASE	AGE	SEX	IT	O	IBD	DX IBD	DX AMOEB	FCS
**1**	35	M	Y	L	Crohn’s	31-01-08	13-02-08	Serpiginous ulcers in the sigmoid colon and rectum
**2**	51	M	N	L	Crohn’s	01-11-12	01-11-14	Ulcers in colon and rectum
**3**	31	F	N	I	Crohn’s	04–14	02–2017	Deep serpiginous ulcers in transverse colon and cecum
**4**	31	F	Y	L	Crohn’s	03–2012	05–2014	Ulcers in rectum and ascending colon
**5**	57	M	Y	L	ulcerative colitis	11–2017	12–2017	Unstructured mucosa in cecum and rectum with deep serpiginous ulcers

IT: Recent international travel (Yes/No)

O: Origin Local/Immigrant

IBD: Inflammatory bowel disease diagnosis

DX IBD: Date of IBD diagnosis

DX AMOEB: Date of amoebic colitis diagnosis

FCS: Results of fibre-optic colonoscopy

HIV-positive cases were more likely to be men (p = 0.009), were less likely to have engaged in prior international travel (p = 0.033) and presented with fewer symptoms compared to HIV-negative subjects (fever p = 0.015, dysentery p = 0.006, abdominal pain p = 0.007, abscess p = 0.067) (Table 3). Nevertheless, the proportion of positive stool tests was higher (p = 0.036).

**Table 3:** Characteristics of the 13 patients diagnosed with both HIV and amoebic colitis.

	N (%)	P value
Male	13 (100%)	0.009
Fever	0 (0%)	0.015
Abdominal pain	4 (30%)	0.007
Dysentery	1 (7%)	0.006
Abscess	0 (0)	0.067
Cysts or trophozoites in stool	13 (100%)	0.036
International travel antecedent	4 (30%)	0.033

Patients with extraintestinal amoebiasis (abscess presence) were less likely to have parasites in stool (p = 0.041) and a greater proportion of positive antibodies in serum; clinically they were more likely to report fever (p < 0.001) and weight loss (p = 0.05).

## Discussion

The data gathered in this retrospective study raise several issues of interest. First of all, as we have noted, in industrialized countries *E*. *histolytica* is non-endemic and mainly restricted to immigrants and returning international travellers. This raises the question as to how infection by *E*. *histolytica* might have taken place in such cases who reported not having engaged in international travel prior to experiencing symptoms. Among patients who did not report travel abroad, all except one were men; most were MSM aged between 30–60 years, of which four had been diagnosed with HIV, while a fifth reported having experienced another sort of sexually transmitted infection. This strongly suggests that *E*. *histolytica* could have been transmitted sexually. Supporting this hypothesis, *E*. *histolytica* has been recognised by other authors as an emerging sexually transmissible pathogen in MSM, causing sporadic outbreaks in countries where it is not endemic [9–11]. For some time now there has also been agreement that patients with HIV infection tend to have a higher prevalence of *E*. *histolytica* infections (but not necessarily at increased risk for developing invasive disease) [12]. Moreover, recent studies have demonstrated that HIV-infected MSM were at significantly higher risk for acquisition of *E*. *histolytica* infection and invasive amoebiasis than other HIV-infected patients, at least in low prevalence countries. Thus, physicians treating MSM with or without HIV infection should be aware of potential *E*. *histolytica* infection, despite the fact that until recently it was nearly always limited to travellers returning from *E*. *histolytica*-endemic regions [13]. In particular, HIV-positive patients (and especially MSM) with chronic diarrhoea should be screened for amoebic colitis even if they have not recently travelled abroad.

Amoebiasis has a great capacity to spread among the MSM population sometimes causing severe cases. This risk may be viewed within the context of other emerging sexually-transmitted enteric infections reported in the EU, hepatitis A being a noteworthy example [14]. Asymptomatic infection should be tested for and treated because of its potential to progress to an invasive disease. Periodic screening using molecular techniques in rectal swabs could prove a useful technique to prevent the spread of infection, especially in MSM populations with high-risk behaviours [15].

A second important issue—this time related to diagnosis—is the difficulty involved in differentiating between intestinal amoebiasis and IBD. This is because the typical symptom of both diseases is subacute diarrhoea leading to weight loss and abdominal pain. Distinguishing these clinically similar diseases is complicated by the possibility that the respective amoebic trophozoites or IBD pathognomonic features will not be seen in biopsies. Concern about this issue is justified by our own data, given that 10% of the cases reviewed here were wrongly diagnosed as IBD, even though diagnosis was carried out at tertiary referral institutions with full diagnostic facilities. In the worst case, a misdiagnosis of amoebic colitis as IBD followed by treatment with corticosteroids may be fatal [16].A recent systematic review concludes that possible infection with *E*. *histolytica* should always be considered prior to the administration of corticosteroids, in particular among patients residing in endemic areas or those with a travel history [17]. Early diagnosis and treatment are essential to avoid progress to fulminant colitis [18]. The screening of amebiasis is an opportunity to additionally screen for *Strongyloides* infection in patients with a history of epidemiological risk, especially among patients taking corticosteroids and/or immunosuppressive treatment considered high risk for hyperinfection syndrome [19].

Invasive amoebae infections show a greater prevalence in patients with IBD when compared to the general population. This suggests that empirical anti-amoebic therapy should be recommended in cases of persistent or relapsing IBD, especially in endemic areas [20,21].

With regard to the diagnosis of amoebic colitis, current techniques include microscopy, antigen detection, serology, molecular techniques, and colonoscopy with histological examination. The best diagnostic results are yielded by the combination of serology or antigen testing, together with identification of the parasite in stool samples or extraintestinal sites. Identification of *E*. *histolytica* in stools by direct wet mount microscopic examination alone can yield a significant proportion of false- positive results [7], with conventional microscopic examination of one a single stool sample showing very low sensitivity (25%). However, sensitivity can be increased to 80% by examining a minimum of three samples on separate days. Furthermore, *E*. *histolytica* is indistinguishable from *E*. *dispar* or *E*. *moshkovskii* (generally considered nonpathogenic amoebas) in direct microscopic examination, making a definitive diagnosis of *E*. *histolytica* difficult. Therefore the facilities to perform at least one more specific assay, particularly an antigen test, must be available in every laboratory [7].

Field studies that have directly compared polymerase chain reaction (PCR) techniques with stool culture or antigen-detection tests for the diagnosis of *E*. *histolytica* infection suggest that these three methods perform equally well [22]. Serum anti-amoebic antibodies have been reported present in up to 90% of patients with symptomatic *E*. *histolytica* infection [23]. However, one drawback of serologic testing is that patients remain positive for many years after infection, making it difficult to distinguish new from past infection in regions of the world where seroprevalence is high. This constitutes further justification for reliance on a combination of approaches for the diagnosis of this disease. According to the literature, the microbiological diagnosis of amoebic colitis can be troubling but, considering our results, diagnosis is best accomplished by the combination of compatible symptoms together with the identification of parasites in stools.

Sigmoidoscopy and/or colonoscopy can also be performed, either to confirm a diagnosis of amoebiasis or to exclude other causes of colitis. These techniques can identify colonic lesions resulting from amoebic dysentery, which range from nonspecific mucosal thickening and inflammation to classic flask-shaped amoebic ulcers. A colonoscopy or sigmoidoscopy can also serve to obtain tissue for histological examination. However, colonoscopy is not recommended as a routine diagnostic approach as it is costly, inconvenient for the patient and risky because intestinal amoebic ulcerations increase the likelihood of perforation during insufflation of air to expand the colon. Other reasons for not doing routine colonoscopy are the expense and inconvenience for patients, when cheaper and easier tests are avaible. The decision to perform these techniques should therefore be based on a high degree of clinical suspicion and be preceded by a thorough epidemiological history including sexual behaviour and travel antecedents [20].

It will have been noted that 16% of the population under study here (eight patients) presented with abscess. In fact, in general 10%-35% of amoebic liver abscess cases report gastrointestinal symptoms [2]. Hepatomegaly is also a typical finding [24].Ultrasonography, abdominal computed tomography and magnetic resonance imaging are all excellent for detecting liver lesions (usually single lesions in the right lobe). Helpful clues to the diagnosis include the presence of epidemiologic risk factors for amoebiasis and the presence of serum anti-amoebic antibodies, positive in 80%-90% of patients.

Finally, with regard to treatment, all *E*. *histolytica* infections should be treated for their potential risk of invasion and further spread. The goal of therapy should consist of both the elimination of trophozoites, which should be treated with a systemic drug such as metronidazole or tinidazole, and the elimination of cysts by means of an intraluminal drug such as paromomycin, diiodohydroxyquin or diloxanide furoate.

Nitroimidazoles, particularly a 10-day course of metronidazole, are the mainstay of therapy for invasive amoebiasis. Nitroimidazoles with longer half-lives (e.g., tinidazole 2 g PO once × 3 days) are better tolerated and allow shorter periods of treatment with a cure rate around 90%. However, amoebic cysts remain in the intestine in as a many as 60% of individuals who receive nitroimidazole, so treatment should be followed by paromomycin or the second-line agent diloxanide furoate to cure luminal infection [25].

One of the main limitations of the study is the absence of a gold standard diagnostic test to diagnose amoebic colitis. Not all available diagnostic tests were applied to all patients and, therefore, it was not possible to estimate the predictive values of each diagnostic technique. Since 90% of *E*.*histolytica* infections are not invasive, if only faecal microscopy is used, it is possible that other causes of colitis are misdiagnosed as amoebic disease. Neverthless the favorable response to the treatment supported the presumptive diagnosis.

In conclusion, amoebic colitis should be assumed in patients with chronic diarrhoea and compatible epidemiological antecedents (immigrants and/or travellers) and MSM, especially if HIV infection is present. These risk factors must be taken into account in any diagnostic approach to inflammatory bowel disease (IBD), and active parasite search should be performed in such cases to rule out misdiagnosis or simultaneous amoebic infection. Also, it is essential that treatment of *E*. *histolytica* infections should include luminal treatment in order to not only avoid relapse but also prevent the spread of the disease.

## References

[1] Bercu TE, Petri WA, Behm JW. Amebic colitis: new insights into pathogenesis and treatment. Curr Gastroenterol Rep 2007; 9:429 17991346 10.1007/s11894-007-0054-8

[2] Haque R, Huston CD, Hugues M, et al Amebiasis. N Engl J Med 2003; 348: 1565 10.1056/NEJMra022710 12700377

[3] Petri WA Jr, Mann BJ, Haque R. The bittersweet interface of parasite and host: lectin-carbohydrate interactions during human invasion by the parasite *Entamoeba histolytica*. Annu Rev Microbiol 2002; 56:39–64. 10.1146/annurev.micro.56.012302.160959 12142490

[4] Pritt BS, Clark CG. Amebiasis. Mayo Clin Proc. 2008; 83(10):1154–1159. 10.4065/83.10.1154 18828976

[5] Adams EB, MacLeod IN. Invasive amebiasis, amebic dysentery and its complications. Medicine (Baltimore) 1977; 56:315–323. 195178 10.1097/00005792-197707000-00003

[6] Stanley SL Jr. Amoebiasis. Lancet 361: 1025–1034. 10.1016/S0140-6736(03)12830-9 12660071

[7] Uslu H, Aktas O, Uyanik MH. Comparison of Various Methods in the diagnosis of *Entamoeba histolytica* in stool and serum speciments. Eurasian J Med. 2016; 48(2): 124–129. 10.5152/eurasianjmed.2015.0074 27551176 PMC4970550

[8] Prathap K, Gilman R. The histopathology of acute intestinal amebiasis: a rectal biopsy study. Am J Pathol 1970; 60: 229–246. 5457212 PMC2032904

[9] Hung CC, Chang SY, Ji DD. *Entamoeba histolytica* infection in men who have sex with men. Lancet Infect Dis 2012; 12(9):729–736. 10.1016/S1473-3099(12)70147-0 22917103

[10] Stark D, van Hal SJ, Matthews G, et al Invasive amebiasis in men who have sex with men, Australia. Emerg Infect Dis. 2008; 14(7):1141–1143. 10.3201/eid1407.080017 18598643 PMC2600324

[11] Weitzel T, Carbera J, Rosas R, et al Enteric multiplex PCR panels: A new diagnostic tool for amoebic liver abscess? New Microbes New Infect. 2017; 18:50–53. 10.1016/j.nmni.2017.05.002 28626584 PMC5460741

[12] Morán P, Ramos F, Ramiro M, et al Infection by human immunodeficiency virus-1 is not a risk factor for amebiasis. Am J Trop Med Hyg 2005; 73:296–300. 16103593

[13] Hung CC, Ji DD, Sun HY, Lee YT, Hsu SY, Chang SY et al Increased Risk for *Entamoeba histolytica* infection and invasive amebiasis in HIV seropositive men who have sex with men in Taiwan (2008). PLOs Negl Trop Dis 2(2): e175 10.1371/journal.pntd.0000175 18301730 PMC2254204

[14] Beebeejaun K, Degala S, Balogun K, Simms I, Woodhall SC, Heinsbroek E, et al Outbreak of hepatitis A associated with men who have sex with men (MSM), England, July 2016 to January 2017. Euro Surveill. 2017; 22(5):30454 10.2807/1560-7917 ES.2017.22.5.30454 . 28183392 PMC5388117

[15] Escolà-Vergé L et al Outbreak of intestinal amoebiasis among men who have sex with men, Barcelona (Spain), October 2016 and January 2017 Euro Surveill 2017; 22(30): pii = 30581. 10.2807/1560-7917. ES. 2017.22.3030581. 28797327 PMC5553055

[16] Hansen LH, Lund C. Amebiasis—A differential diagnosis from inflammatory bowel disease. Ugeskr Laeger 1998; 160(38): 5514–5515. 9763927

[17] Shirley DB, Moonah S. Fulminant amebic colitis after corticosteroid therapy: A systematic Review. PLOS Negl Trop Dis 10(7): e0004879 10.1371/journal.pntd.0004879 27467600 PMC4965027

[18] Wong Toh Yoon E, Sumii M. Severe amoebic colitis in an HIV-infected male patient. BMJ Case Rep 2016 10.1136/bcr-2016-218570 28039352 PMC5237780

[19] Keiser PB. Nutman TB: *Strongyloides stercoralis* in the Immunocompromised population. Clin Microbiol Rev. 2004;17:208–1 10.1128/CMR.17.1.208-217.2004 14726461 PMC321465

[20] Babic E, Bevanda M, Mimica M, Karin M, Volaric M et al Prevalence of amebiasis in inflammatory bowel disease in University Clinical Hospital Mostar. Springerplus 2016 15; 5(1): 1586 10.1186/s40064-016-3261-7 eCollection 2016. 27652159 PMC5025403

[21] Petridou C, Al-Badri A, Dua A, Dryden M, Saeed K. Learning points from a case of severe amoebic colitis. Le infezioni in Medicina 2017; 3:281–284. 28956549

[22] Evangelopoulos A, Legakis N, Vakalis N. Microscopy, PCR and ELISA applied to the epidemiology of amoebiasis in Greece. Parasitol Int 2001; 50:185–189. 11595575 10.1016/s1383-5769(01)00078-2

[23] Haque R, Ali IKM, Akhter S, Petri WA Jr. Comparison of PCR, isoenzyme analysis, and antigen detection for diagnosis of *Entamoeba histolytica* infection. J Clin Microbiol 1998; 36:449–452. 9466756 10.1128/jcm.36.2.449-452.1998 PMC104557

[24] Adams EB, MacLeod IN. Invasive amebiasis. Amebic liver abscess and its complications. Medicine (Baltimore) 1977; 56:325–34. 875719 10.1097/00005792-197707000-00004

[25] Drugs for Parasitic Infections, 3rd Ed., The Medical Letter 2013 (New Rochelle, NY).

